# Supplementary material for: Case finding of tuberculosis among mining communities in Ghana
Source: PLoS One. 2021 Mar 18;16(3):e0248718. doi: 10.1371/journal.pone.0248718 (PMC7971527; doi:10.1371/journal.pone.0248718)
Supplement: Attachment — Submitted filename: PONE-D-20-34085_reviewed by Dr. Shamim. M..pdf [file pone.0248718.s002.pdf]

PLOS ONE

Case finding of tuberculosis among mining communities in Ghana

--Manuscript Draft--

**Manuscript Number:**	PONE-D-20-34085
**Article Type:**	Research Article
**Full Title:**	Case finding of tuberculosis among mining communities in Ghana
**Short Title:**	TB case finding in Ghana mining communities
**Corresponding Author:**	Sally-Ann Ohene<br>World Health Organization<br>Accra, GHANA
**Keywords:**	Ghana; tuberculosis; mining communities
**Abstract:**	Background<br><br>Data on active TB case finding activities among mining communities is limited. The study assessed the yield of TB cases from the TB screening activities among mining communities, in Ghana, the factors associated with TB in these communities and the correlation between the screening methods and a diagnosis of TB.<br><br>Methods<br><br>We conducted secondary data analyses of NTP program data collected from TB case finding activities using symptom screening and mobile X-ray implemented in hard to reach mining communities. Yield of TB cases, number needed to screen (NNS) and the number needed to test (NNT) to detect a TB case were assessed and logistic regression were conducted to assess factors associated with TB. The performance of screening methods chest X-ray and symptoms in the detection of TB cases was also evaluated.<br><br>Results<br><br>In total 10,441 people from 78 communities in 24 districts were screened, 55% were female and 60% (6,296) were in the aged 25 to 54 years. Ninety-five TB cases were identified, 910 TB cases per 100,000 population screened; 5.6% of the TB cases were rifampicin resistant. Being male (aOR 5.96, 95% CI 3.25–10.92, P < 0.001), a miner (aOR 2.70, 95% CI 1.47–4.96, P = 0.001) and age group 35 to 54 years (aOR 2.27, 95% CI 1.35–3.84, P =0.002) were risk factors for TB. NNS and NNT were 110 and 24 respectively. All symptoms correlated with X-ray suggestive of TB; cough of any duration had the strongest association (aOR 10.85, 95% CI 8.77–13.42, P < 0.001). Cough was most sensitive for a diagnosis of TB; sensitivity of 86.3% (95% CI 79.4 - 93.2) followed by X-ray, sensitivity 81.1% (95% CI 71.7-88.4). The specificities of the symptoms and X-rays ranged from 80.2% (cough) to 97.3% (sputum).<br><br>Conclusion<br><br>The high risk of TB in the mining communities and in miners in this study reinforces the need to target these populations with outreach programs particularly in hard to reach areas. The diagnostic value of cough highlights the usefulness of symptom screening in this population that may be harnessed even in the absence of X-ray to identify those suspected to have TB for further evaluation.
**Order of Authors:**	Sally-Ann Ohene
	Frank Bonsu
	Yaw Adusi-Poku
	Francisca Dzata
	Mirjam Bakker

Powered by Editorial Manager® and ProduXion Manager® from Aries Systems Corporation

Additional Information:	
**Question**	**Response**
**Financial Disclosure**<br><br>Enter a financial disclosure statement that describes the sources of funding for the work included in this submission. Review the submission guidelines for detailed requirements. View published research articles from *PLOS ONE* for specific examples.<br><br>This statement is required for submission and **will appear in the published article** if the submission is accepted. Please make sure it is accurate.<br><br>**Unfunded studies**<br>Enter: *The author(s) received no specific funding for this work.*<br><br>**Funded studies**<br>Enter a statement with the following details:<br>• Initials of the authors who received each award<br>• Grant numbers awarded to each author<br>• The full name of each funder<br>• URL of each funder website<br>• Did the sponsors or funders play any role in the study design, data collection and analysis, decision to publish, or preparation of the manuscript?<br>• **NO** - Include this sentence at the end of your statement: *The funders had no role in study design, data collection and analysis, decision to publish, or preparation of the manuscript.*<br>• **YES** - Specify the role(s) played.<br><br>* typeset	The authors received no specific funding for this work
**Competing Interests**<br><br>Use the instructions below to enter a competing interest statement for this submission. On behalf of all authors, disclose any competing interests that could be perceived to bias this work—acknowledging all financial support and any other relevant financial or non-financial competing interests.	The authors have declared that no competing interests exist.

This statement **will appear in the published article** if the submission is accepted. Please make sure it is accurate. View published research articles from *PLOS ONE* for specific examples.<br><br>**NO authors have competing interests**<br><br>Enter: *The authors have declared that no competing interests exist.*<br><br>**Authors with competing interests**<br><br>Enter competing interest details beginning with this statement:<br><br>*I have read the journal's policy and the authors of this manuscript have the following competing interests: [insert competing interests here]*<br><br>* typeset	
**Ethics Statement**<br><br>Enter an ethics statement for this submission. This statement is required if the study involved:<br><br>• Human participants<br>• Human specimens or tissue<br>• Vertebrate animals or cephalopods<br>• Vertebrate embryos or tissues<br>• Field research<br><br>Write "N/A" if the submission does not require an ethics statement.<br><br>General guidance is provided below. Consult the submission guidelines for detailed instructions. **Make sure that all information entered here is included in the Methods section of the manuscript.**	N/A

Powered by Editorial Manager® and ProduXion Manager® from Aries Systems Corporation

**Format for specific study types**<br><br>**Human Subject Research (involving human participants and/or tissue)**<br>• Give the name of the institutional review board or ethics committee that approved the study<br>• Include the approval number and/or a statement indicating approval of this research<br>• Indicate the form of consent obtained (written/oral) or the reason that consent was not obtained (e.g. the data were analyzed anonymously)<br><br>**Animal Research (involving vertebrate animals, embryos or tissues)**<br>• Provide the name of the Institutional Animal Care and Use Committee (IACUC) or other relevant ethics board that reviewed the study protocol, and indicate whether they approved this research or granted a formal waiver of ethical approval<br>• Include an approval number if one was obtained<br>• If the study involved *non-human primates*, add *additional details* about animal welfare and steps taken to ameliorate suffering<br>• If anesthesia, euthanasia, or any kind of animal sacrifice is part of the study, include briefly which substances and/or methods were applied<br><br>**Field Research**<br><br>Include the following details if this study involves the collection of plant, animal, or other materials from a natural setting:<br>• Field permit number<br>• Name of the institution or relevant body that granted permission	
**Data Availability**<br><br>Authors are required to make all data underlying the findings described fully available, without restriction, and from the time of publication. PLOS allows rare exceptions to address legal and ethical concerns. See the PLOS Data Policy and FAQ for detailed information.	Yes - all data are fully available without restriction

Powered by Editorial Manager® and ProduXion Manager® from Aries Systems Corporation

A Data Availability Statement describing where the data can be found is required at submission. Your answers to this question constitute the Data Availability Statement and **will be published in the article**, if accepted.<br><br>**Important:** Stating 'data available on request from the author' is not sufficient. If your data are only available upon request, select 'No' for the first question and explain your exceptional situation in the text box.<br><br>Do the authors confirm that all data underlying the findings described in their manuscript are fully available without restriction?	
**Describe where the data may be found in full sentences. If you are copying our sample text, replace any instances of XXX with the appropriate details.**<br><br>• If the data are **held or will be held in a public repository**, include URLs, accession numbers or DOIs. If this information will only be available after acceptance, indicate this by ticking the box below. For example: *All XXX files are available from the XXX database (accession number(s) XXX, XXX.).*<br>• If the data are all contained **within the manuscript and/or Supporting Information files**, enter the following: *All relevant data are within the manuscript and its Supporting Information files.*<br>• If neither of these applies but you are able to provide **details of access elsewhere**, with or without limitations, please do so. For example:<br><br>*Data cannot be shared publicly because of [XXX]. Data are available from the XXX Institutional Data Access / Ethics Committee (contact via XXX) for researchers who meet the criteria for access to confidential data.*<br><br>*The data underlying the results presented in the study are available from (include the name of the third party*	All relevant data are within the manuscript and its Supporting Information files

and contact information or URL).<br>• This text is appropriate if the data are owned by a third party and authors do not have permission to share the data.<br><br>* typeset	
Additional data availability information:	

Powered by Editorial Manager® and ProduXion Manager® from Aries Systems Corporation

Manuscript

Click here to access/download;Manuscript;TB case finding mining communities 30 Oct 20.doc

Case finding of tuberculosis among mining communities in Ghana

Sally-Ann Ohene[1*], Frank Bonsu[2], Yaw Adusi-Poku[2], Francisca Dzata[2], Mirjam Bakker[3]

[1]World Health Organization Country Office, Accra, Ghana

[2] National Tuberculosis Control Program, Ghana Health Service, Accra, Ghana

[3]KIT Health, Royal Tropical Institute (KIT), Amsterdam, The Netherlands

* Corresponding author
Email: salohene@yahoo.com (SAO)
Orcid ID: 0000-0002-7511-5270

Abstract

Background

Data on active TB case finding activities among mining communities is limited. The study assessed the yield of TB cases from the TB screening activities among mining communities, in Ghana, the factors associated with TB in these communities and the correlation between the screening methods and a diagnosis of TB.

Methods

We conducted secondary data analyses of NTP program data collected from TB case finding activities using symptom screening and mobile X-ray implemented in hard to reach mining communities. Yield of TB cases, number needed to screen (NNS) and the number needed to test (NNT) to detect a TB case were assessed and logistic regression were conducted to assess factors associated with TB. The performance of screening methods chest X-ray and symptoms in the detection of TB cases was also evaluated.

Results

In total 10,441 people from 78 communities in 24 districts were screened, 55% were female and 60% (6,296) were in the aged 25 to 54 years. Ninety-five TB cases were identified, 910 TB cases per 100,000 population screened; 5.6% of the TB cases were rifampicin resistant. Being male (aOR 5.96, 95% CI 3.25–10.92, $P < 0.001$), a miner (aOR 2.70, 95% CI 1.47–4.96, $P = 0.001$) and age group 35 to 54 years (aOR 2.27, 95% CI 1.35–3.84, $P =0.002$) were risk factors for TB. NNS and NNT were 110 and 24 respectively. All symptoms correlated with X-ray suggestive of TB; cough of any duration had the strongest association (aOR 10.85, 95% CI 8.77–13.42, $P < 0.001$). Cough was most sensitive for a diagnosis of TB; sensitivity of 86.3% (95% CI 79.4 -93.2) followed by X-ray, sensitivity 81.1% (95% CI 71.7-

88.4). The specificities of the symptoms and X-rays ranged from 80.2% (cough) to 97.3% (sputum).

Conclusion

The high risk of TB in the mining communities and in miners in this study reinforces the need to target these populations with outreach programs particularly in hard to reach areas. The diagnostic value of cough highlights the usefulness of symptom screening in this population that may be harnessed even in the absence of X-ray to identify those suspected to have TB for further evaluation.

Key words: Ghana, tuberculosis, mining communities

Introduction

Tuberculosis (TB) remains the leading cause of death from an infectious agent in the twenty first century despite being curable with antimicrobial drugs [1]. An estimated 10 million people developed TB in 2018, while about 1.2 million deaths occurred as a result of the disease [1]. The WHO African Region has the highest TB incidence rate of 231 per 100,000 population [1]. Ghana classified a high HIV/TB burden, like several countries in the African region, grapples with TB control activities [2]. The TB cases notified annually in Ghana averaged 14, 500 TB cases in the years preceding the national prevalence survey conducted in 2013 and thereafter [2,3]. The prevalence survey findings however suggested that people being detected with TB represent just about 21% of actual cases highlighting the need to adopt different strategies to identify more cases. [2] The END TB Strategy of the World Health Organization emphasizes early diagnosis of TB that involves systematic screening of contacts and high-risk groups, universal drug–susceptibility testing and standardized treatment of TB and drug-resistant TB forms. [4] Early case detection coupled with a high treatment success leads to a cure of infectious TB cases and cuts the risk of transmission ultimately reducing the burden of TB. This has led to several countries adopting various strategies to improve case detection. [5-7]

Ghana is one such country in which a 2013 comprehensive review of the Ghana National TB Program highlighted the need to improve access to TB prevention, treatment, care and support services for key affected populations including PLHIV, children, diabetics, and miners or those exposed to silica [8]. Consequently, the National TB Health Sector Strategic Plan for Ghana (2015-2020) clearly identified TB case detection among mining populations as one of these key populations for intervention [2].

High risk populations such as miners especially those engaged in illegal mining activities may however be in hard to reach areas where they may encounter challenges accessing health

services including those for diagnosing TB [9,10]. Strategies such as deployment of mobile teams proved to be useful in identifying people with TB in such hard to reach populations and mining communities [10-12]. The implementation of active case finding (ACF) activities using mobile teams in hard to reach mining communities in Myanmar identified significantly higher TB prevalence in these townships than in the rest of the country [11]. In a study conducted among South African miners, death from TB was more than 2 times higher among those with TB diagnosed through passive case finding than those diagnosed through active screening exercises [13].

The Ghana NTP with funding from the Global Fund supported interventions to improve TB case finding among mining communities in the country [14]. Given the limited data on active TB case finding activities among mining communities in Ghana, the aim of the study was to assess the yield of TB cases from the TB screening activities among mining communities, the factors associated with TB in these communities and the correlation between the screening methods and a diagnosis of TB.

Methods

Study Design and Setting

The study was cross-sectional consisting of secondary data analyses of routine NTP program data collected from TB case finding activities implemented in selected mining communities from May 2017 to January 2018. These activities were undertaken as part of NTP's efforts to make available TB screening services to vulnerable populations in hard to reach areas. [2] Having already undertaken passive TB control activities in mining areas in Ashanti, Brong Ahafo and Western regions (collective estimated population of 11,200,955 in 2017), the NTP in consultation with the respective Regional Health Directorates (RHD) selected 3, 6 and 12 districts respectively from each region with intense mining activities.

These 21 districts consisted of an estimated total population of 2,120,660 in 2017 with TB notification rates ranging from 37 to 394/100,000 population. In consultation with the RHD and District Health Directorates (DHD), 78 communities were selected from these districts for TB screening on the basis of TB case burden and being hard to reach thus with difficulty accessing health services.

The NTP deployed mobile teams consisting of a team leader to superintend the screening team, field coordinator to coordinate the field activities, radiographer to operate the digital X-ray machine, a physician-assistant to read the digital X-ray information technologist to maintain network connectivity for the transmission and storage of X-ray images and data, engineer to maintain the X –ray equipment and electrical generators, support team members to conduct screening and collect sputum samples and drivers for the digital X-ray van and vehicle to transport the team and other logistics. A week before the arrival of the team to the community, the district TB focal person in collaboration with the community volunteers undertook community mobilization using various methods including house to house sensitization and community radio announcements informing the community member of the upcoming free TB screening exercise, the date and the place where the team will be located.

Study population and data collection

All community members 15 years and above in the designated communities were eligible to be included in the screening exercise though the dataset showed that a handful of children less than 15 years were also screened. On the day of the exercise, community members voluntarily presenting themselves at the location of the mobile team were registered and their socio-demographic information recorded. A TB symptom screening was administered using a questionnaire inquiring about the presence or absence of cough, fever, sputum production, chest pain, weight loss, night sweats and fever. With the exception of the following; pregnant

or possibly pregnant women, those who for one reason or the other could not take or declined taking the chest X-ray, everyone's X-ray was taken using the digital X-ray machine. A physician assistant trained to read digital X-ray images categorized the X-ray finding into 3 groups: normal, abnormal suggestive of TB and abnormal but unlikely to be TB. Those reported to have an abnormal X-ray suggestive of TB, those admitting having a cough of 2 weeks or any duration with at least one other symptom were presumed to have TB and were requested to produce 1 spot sample of sputum. Also included in the presumed TB group and asked to produce sputum for testing were those unable to have an X-ray taken as indicated above and HIV positive individuals. The samples were transported to designated laboratories with a GeneXpert machine for testing. The GeneXpert results were reported as Mycobacterium tuberculosis (MTB) not detected, MTB detected rifampicin sensitive and MTB detected rifampicin resistance detected. The field coordinator followed up on the results and arranged for those found to have TB to be registered at the district level to start TB treatment. The analyses of the treatment outcomes of these TB patients were beyond the scope of this paper.

Statistical analyses

The dataset for the analyses consisted of the population that was screened. Descriptive analyses were conducted for the following variables district, age, sex, occupation, X-ray reading, presence of TB symptoms and GeneXpert results according to region. Among the population screened, those with TB were compared with those without TB using Fisher's exact test. The prevalence of TB was calculated and so were the number needed to screen (NNS) and the number needed to test (NNT) to detect a TB case. Univariate logistic regression was initially conducted to assess factors associated with TB. Given the few characteristics being examined, all variables were then entered into a multivariable logistic regression model. Odds ratio and 95% confidence (95%CI) interval were assessed. The

association between symptoms and having X-ray suggestive of TB was also determined by assessing Cohen Kappa's coefficient as well as multivariable logistics regression. Finally, the performance of screening methods chest X-ray and symptoms in the detection of TB cases was also evaluated. In the sensitivity analysis all those not tested were regarded not to have TB. STATA version 13 was used for statistical analyses and $p<0.05$ was set as the level of significance. The Ghana Health Service Ethical Review Committee gave ethical approval for the conduct of the study.

RESULTS

Demographic characteristics and yield of TB among screened population

From among the 3 regions Brong Ahafo, Ashanti and Western, a total of 10,441 people from 78 communities in 21 districts were screened. On the average 226 people were screened a day. The number of people screened per community ranged from 39 to 202 while for the districts, the numbers screened ranged from 282 to 826. Overall 55% were female and 60% (6,296) were in the age group 25 to 54 years. There were 23 children (0.2%) less than 15 years. About 42% of the people screened were in the agriculture sector while miners comprised 8.5%. The demographic characteristics of the study population by region are presented in Table 1.

Table 1 Demographic characteristics of person screened in mining communities in Ashanti, Brong Ahafo And Western Regions May 2017 to January 2018

	**Ashanti N= 1896**	**Brong Ahafo N=3252**	**Western N=5293**

Districts	3	6	12
**Communities**	14	24	40
**Age (years)**			
<15	0	13 (0.4)	10 (0.2)
15 – 24	240 (12.7)	357 (11.0)	686 (13.0)
25 – 34	368 (19.4)	676 (20.8)	1,225 (23.1)
35 – 44	351 (18.5)	653 (20.1)	1,095 (20.7)
45 – 54	350 (18.5)	581 (17.9)	997 (18.8)
55 – 64	312 (16.5)	433 (13.3)	650 (12.3)
65+	272 (14.3)	536 (16.5)	620 (11.7)
Missing	3 (0.2)	3 (0.1)	9 (0.2)
**Sex**			
Male	882 (47.0)	1481 (45.5)	2,268 (42.9)
Female	995 (52.5)	1769 (54.4)	3,018 (57.0)
Missing	9 (0.5)	2 (0.1)	7 (0.1)
**Occupation**			
Agriculture	771 (37.5)	1639 (50.4)	1,911 (36.1)
Professional	130 (6.9)	242 (7.4)	250 (4.7)
Miner	103 (5.4)	278 (8.6)	486 (9.2)
Sales and services	497 (26.2)	587 (18.1)	1,496 (28.3)
Manual labour	114 (6.0)	281 (8.6)	447 (8.5)
Unemployed	319 (16.8)	182 (5.6)	489 (9.2)
Missing	22 (1.2)	43 (1.3)	214 (4.0)
**Symptoms**			
Cough any duration	484 (25.5)	575 (17.7)	1,074 (20.3)
Sputum	2 (0.1)	232 (7.1)	50 (0.9)
Chest pain	527 (27.8)	336 (10.3)	772 (14.6)
Weight loss	442 (23.3)	154 (4.7)	644 (12.2)
Night sweats	309 (16.3)	206 (6.3)	518 (9.8)
Fever	251 (13.2)	57 (1.8)	399 (7.5)
No symptoms	1037 (54.7)	2388 (73.4)	3697 (69.8)
**X-ray Results**			
Normal	1,082 (57.1)	2937 (90.3)	3,572 (67.5)
Abnormal TB	387 (20.4)	213 (6.6)	848 (16.0)
Abnormal Non-TB	413 (21.8)	79 (2.4)	798 (15.1)
Missing	14 (0.7)	23 (0.7)	75 (1.4)

About half of those screened 50.7% were from Western Region. Ashanti Region had the smallest proportion of miners 5.4% while 50.4% of those screened in Brong Ahafo were in the agriculture sector. The districts in which the TB case finding activities were undertaken are shown in Figure 1.

Fig 1. Map of Ghana showing districts in which case finding activities were undertaken in selected mining communities from May 2017 to January 2018. Fig 1 legend shows the range of the number of TB cases detected per 1000 persons screened from the TB case finding exercise in the respective districts

Figure 2 shows the algorithm of the overall TB screening process and the yield.

Fig 2. Flow chart of TB case finding screening exercise undertaken in mining communities in 21 districts in Ghana from May 2017 to January 2018

Among those who had a chest X-ray done (10,329), almost 14% (1,448) were reported to have findings suggestive of TB. About 23% of those screened were identified as presumed TB cases. The 95 TB cases identified out of the 10,441 people screened was equivalent to 910 TB cases per 100,000 population screened. Out of the 78 communities, thirty-seven (47%) did not have any TB cases detected, fourteen communities had 1 TB case, sixteen had 2, two had 3 TB cases detected. Six communities had 4 TB cases, two had 5 and 1 had 9 TB cases detected. The community prevalence varied between 0 and 6.8%. No TB cases were identified in 3 districts out of the 21 districts undertaking TB case finding activities. Ashanti recorded the highest yield of TB cases among those screened 1.32%, compared to Western 1.11% and Brong Ahafo 0.34%. Table 2 shows the yield of TB cases by regions following TB case finding exercises undertaken in selected mining communities in selected districts

Table 2 Yield of TB cases from TB case finding exercise undertaken in 3 regions in Ghana May 2017 to January 2018

Regions	Ashanti	Brong Ahafo	Western	Total
**Number screened**	1896	3252	5293	10441
**Presumptive TB (% out of screened)**	444 (23.4)	536 (16.5)	1,409 (26.0)	2,389 (22.9)
**Number tested (% of presumptive TB)**	441 (99.3)	401 (74.8)	1408 (99.9)	2250 (94.2)
**Number bacteriologically positive (% out of tested)**	25 (5.6%)	11 (2.8%)	59 (4.2)	95 (4.2)
**Number rifampicin resistant (% out of TB cases)**	1 (4%)	1 (9.1%)	3 (5.1)	5 (5.3)
**NNT (Number needed to test)**	18	36	24	24
**NNS (Number needed to screen)**	76	296	90	110

Western Region accounted for 62% (59) of the TB cases while 26% (25) were from Ashanti Region. Males made up 84% (80) of the total TB cases, 70% of those with TB were in the age range of 25 to 54 years and miners had the highest prevalence of TB 2.65% compared to 1.41% among those engaged in skilled manual labour and 0.68% among those involved in the

agriculture sector. The overall TB prevalence among non-miners was 0.64%. Five of the TB cases had rifampicin resistant TB. All 5 were male in the 35 to 44 year age group with 2 of them being miners from Western Region giving a rate of 8.7% rifampicin resistant TB among the miners with TB.

Factors associated with TB

In the bivariate analyses shown in Table 3, there was significant association between sex and occupation and TB but not age.

Table 3. Bivariate analyses comparing TB and non-TB cases by demographic variables among persons screened in selected mining communities in Ghana May 2017 to January 2018

	**Ashanti N=1896**			**Brong Ahafo N=3252**			**Western N=5293**		
	**No TB**	**TB**		**No TB**	**TB**		**No TB**	**TB**	
	**N=1871**	**N=25**	**p**	**N=3241**	**N=11**	**p**	**N=5234**	**N=59**	**p**
**Age Years)**									
<15	0	0		13 (0.4)	0		10 (0.2)	0	
15 – 24	240 (12.8)	0		357 (11.0)	0		681 (13.0)	5 (8.5)	
25 – 34	361 (19.3)	7 (28.0)		674 (20.8)	2 (18.2)		1212 (23.2)	13 (22.0)	
35 – 44	345 (18.4)	6 (24.0)	0.35	650 (20.1)	3 (27.3)	0.86	1079 (20.6)	16 (27.1)	0.497
45 – 54	346(18.5)	4 16.0)		579 (17.9)	2 (18.2)		983 (18.8)	14 (23.7)	
55 – 64	307(16.4)	5 (20.0)		432 (13.3)	1 (9.1)		642 (12.3)	8 (13.6)	
65+	269 (14.4)	0		533 (16.4)	3 (27.3)		618 (11.8)	3 (5.1)	
Missing	3 (0.2)			3 (0.1)	0		9 (0.2)	0	
**Sex**									
Male	868 (46.4)	24 (96.0)		1472 (45.4)	9 (82.8)		2221 (42.4)	47 (79.7)	
Female	994 (53.1)	1 (4.0)	< 0.001	1767 (54.5)	2 (18.2)	0.035	3006 (57.4)	12 (20.3)	<0.001
Missing	9 (0.5)	0		2 (0.1)	0		7 (0.1)	0	
**Occupation**									
Agriculture	700 (37.4)	11 (44.0)		1636 (50.5)	3 (27.3)		1896 (36.2)	15 (25.4)	
Professional	130 (7.0)	0		242 (7.5)	0		250 (0.8)	0	
Miner	98 (5.0)	5 (20.0)		276 (8.5)	2 (18.2)		470 (9.0)	16 (27.1)	
Sales and services	495 (26.5)	2 (8.0)	0.025	586 (18.1)	1 (9.1)	p<0.001	1487 (28.4)	9 (15.3)	<0.001
Manual lab	112 (6.0)	2 (8.0)		278 (8.6)	3 (27.3)		442 (8.4)	5 (8.5)	
Unemployed	314 (16.8)	5 (20.0)		182 (5.6)	0		476 (9.1)	13 (22.0)	
Missing	22 (1.2)	0		41 (1.3)	2 (18.2)		213 (4.1)	1 (1.7)	

Those with TB were more likely to be male, compared to females. In the Ashanti Region, all but 1 of the 25 TB case identified were male. Even though miners formed 8 % of those screened, 25% of those with TB were miners with a range of 18% in Brong Ahafo to 27% in Western Region.

In univariate analyses, shown in Table 4, being a male, miner and unemployed were risk factors for having TB as was coming from Western and Ashanti regions. In multivariate

analyses also in Table 4, being in the age group 35 to 54 years became a risk factor for TB, (OR [aOR] 2.27, 95% CI 1.35–3.84, P =0.002). Being male, (OR [aOR] 5.96, 95% CI 3.25–10.92, P < 0.001) miners (OR [aOR] 2.70, 95% CI 1.47–4.96, P = 0.001), unemployed (OR [aOR] 3.15, 95% CI 1.66–5.95, P < 0.001) and coming from Ashanti (OR [aOR] 4.12, 95% CI 1.89–8.98, P < 0.001) and Western Regions (OR [aOR] 3.84, 95% CI 1.90–7.80, P < 0.001) were risk factors for having TB.

Table 4. Logistic regression analyses of factors associated with TB diagnosis N=10,441

	Univariate OR (%CI)	p	Multivariate aOR (95CI)	p
**Age (years)**				
<35	1		1	
35 –54	1.49 (0.92 - 2.40)	0.106	2.27 (1.35 - 3.84)	0.002
≥55	1.08 (0.62 - 1.89)	0.789	1.82 (0.98 - 3.39)	0.055
**Sex**				
Female	1		1	
Male	6.74 (3.88-11.72)	<0.001	5.96 (3.25-10.92)	<0.001
**Occupation**				
Agriculture	1		1	
Professional	-		-	
Miner	3.98 (2.29 - 6.91)	<0.001	2.70 (1.47 - 4.96)	0.001
Sales and services	0.68 (0.35 - 1.34)	0.266	1.12 (0.55 - 2.29)	0.751
Manual labour	1.75 (0.85 – 3.61)	0.127	1.08 (0.51 - 2.30)	0.738
Unemployed	2.70 (1.49 - 4.89)	0.001	3.15 (1.66 – 5.95)	<0.001
**Region**				
Brong Ahafo	1		1	1
Ashanti	3.94 (1.93 - 8.02)	<0.001	4.12 (1.89 – 8.98)	<0.001
Western	3.32 (1.74 - 6.33)	<0.001	3.84 (1.90 – 7.80)	<0.001

OR – Odds ratio, aOR – adjusted odds ratio

Association between TB symptoms and X-ray suggestive of TB and TB diagnosis

In the analyses to assess the correlation between the symptoms and X-ray, shown in Table 5, each of the symptoms, cough of any duration, sputum, chest pain, weight loss, night sweats and fever was found to be significantly associated with X-ray suggestive of TB with cough ranking the highest with Kappa Coefficient of 0.46. Findings were similar in the multivariate analyses controlling for sex and age.

Table 5. Correlation between symptoms and X-ray suggestive of TB using Kappa Coefficient and multivariate logistic regression

	X-ray suggestive of TB N=1,448	X-ray not suggestive of TB N=8,881	kappa	Z	p	Multivariate aOR (95%CI)	p
**Cough any duration**							

Yes	988 (68.2)	1,122 (12.6)				1	
No	460 (31.8)	7,759 (87.4)	0.4667	48.66	<0.001	10.85 (8.77– 13.42)	<0.001
**Sputum**							
Yes	146 (10.1)	135 (1.5)				1	
No	1,302 (89.9)	8,746 (98.5)	0.1292	18.57	<0.001	2.15 (1.60 – 2.90)	<0.001
**Chest pain**							
Yes	686 (47.4)	939 (10.6)				1	
No	762 (52.6)	7,942 (89.4)	0.3501	35.66	<0.001	2.32 (1.96 – 2.75)	<0.001
**Weight loss**							
Yes	612 (42.3)	625 (7.0)				1	
No	836 (57.7)	8,256 (93.0)	0.3752	38.28	<0.001	3.34 (2.80 – 3.98)	<0.001
**Night sweats**							
Yes	488 (33.7)	541 (6.1)				1	
No	960 (66.3)	8,340 (93.9)	0.3141	32.53	<0.001	2.10 (1.74 – 2.54)	<0.001
**Fever**							
Yes	372 (25.7)	334 (3.8)				1	
No	1,076 (74.3)	8,547 (96.2)	0.2792	30.66	<0.001	2.18 (1.77 – 2.69)	<0.001

95%CI – 95% Confidence Interval, aOR Adjusted Odds Ratio

All symptoms correlated with X-ray suggestive of TB with cough of any duration having the strongest association (OR [aOR] 10.85, 95% CI 8.77–13.42, P < 0.001). Cough was also the most sensitive symptom for a diagnosis of TB at a sensitivity of 86.3% (95% CI 79.4 -93.2) followed by weight loss 67.4 % (95% CI 57.0-76.6) and chest pain 63.2% (95% CI 52.6-72.8) with sensitivities that were overlapping Table 6.

Table 6. Performance of Sensitivity and specificity of symptoms and X-ray for diagnosis of TB

	TB cases	No TB	Sensitivity (95%CI)	Specificity (95%CI)	PPV (95%CI)	AUC
**Cough any duration**						
Yes	82	2,051	86.3 (77.7 -92.5)	80.2 (79.4-80.9)	3.8 (3.1-4.8)	0.83 (0.80-0.87)
No	13	8,295				
**Sputum**						
Yes	8	276	8.4 (3.7-15.9)	97.3 (97.0-97.6)	2.8 (1.2-5.5)	0.53 (0.50-0.56)
No	87	10,070				
**Chest pain**						
Yes	60	1,575	63.2 (52.6-72.8)	84.8 (84.1-85.5)	3.7 (2.8-4.7)	0.74 (0.69-0.79)

No	35	8,771				
**Weight loss**						
Yes	64	1,176	67.4 (57.0-76.6)	88.6 (88.0-89.2)	5.2 (4.0-6.5)	0.78 (0.73-0.83)
No	31	9,170				
**Night sweats**						
Yes	34	999	35.8 (26.2-46.3)	90.3 (89.8-90.9)	3.3 (2.3-4.6)	0.63 (0.58-0.68)
No	61	9,347				
**Fever**						
Yes	35	672	36.8 (27.2-47.4)	93.5 (93.0-94.0)	4.9 (3.5-6.8)	0.65 (0.60-0.70)
No	60	9,674				
**X-ray**						
Yes	77	1,371	81.1 (71.7-88.4)	86.6 (85.9-87.3)	5.3 (4.2-6.5)	0.84 (0.80-0.88)
No	18	8,863				
**Cough and X-ray**						
Yes	90	2,503	94.7 (88.1-98.3)	75.8 (75.0-76.6)	3.5 (2.8-4.3)	0.85 (0.83-0.88)
No	5	7,843				

95%CI – 95% Confidence Interval, PPV – Positive predictive value, AUC – Area under the curve

Sputum production had the least sensitivity 8.4% (95% CI 3.7-15.9). The sensitivity of X-ray in the diagnosis of TB 81.1% (95% CI 71.7-88.4) ranked after that of cough but was comparable. The combination of cough and chest X-ray had the highest sensitivity 94.7 (95% CI). The specificities of the symptoms and X-rays ranged from 80.2% (cough) to 97.3% (sputum). The positive predictive values of all the symptoms and X-ray suggestive of TB were all low <7%.

Discussion

Miners and the communities in which they live are identified as being at higher risk for TB than the general population [15]. Forging ahead with the End TB Strategy, responsive programs for such key populations require data that will inform and guide stakeholders in the appropriate planning and delivering of services [4,16]. In this study, which is the first to the best of our knowledge to highlight on active TB case finding among mining communities in Ghana using mobile X-ray and symptom screening, the overall rate of TB among the people screened was found to be 910 per 100,000 people screened with 5.3% of the TB cases having rifampicin resistant TB. The NNS and NNT were 110 and 24 respectively. Risk factors for TB were being male, a miner and location in Ashanti and Western Regions. Cough regardless of duration correlated with X-ray findings suggestive of TB and a diagnosis of TB.

The prevalence of TB among the communities screened in this study was more than two and half times what was reported for the general population in Ghana in the TB prevalence survey (356 bacteriologically confirmed TB per 100,000) re-iterating the data on the higher risk of TB among mining communities [9,15,17]. The overall community TB prevalence was however lower than has been reported in other mining communities such as the copper belt in Zambia (1.2%) and mining communities in Myanmar (2.7%) [11,18]. The TB prevalence among the miners in our study (2.65%), while also lower than that cited in other studies in South Africa (5.4%) and Zambia (9.5%), was higher than that in miners in Myanmar (1.7%) [11,18,19]. These differences may be related to several factors including varying methods for diagnosing TB in the miners such as sputum microscopy as opposed to GeneXpert, the type of mining, the exposure to silica dust and the duration of exposure to mining conditions [18]. In the Zambia study, study participants were underground miners in copper mines while the miners in our study were likely involved in gold and other mineral exploration using different mining methods ranging from surface, to underground mining [18, 20-22].

The rate of rifampicin resistance found among our study population (5.3%) was slightly higher than the 4.3% drug resistance recorded among South African gold miners and their dependents but much higher than the 1.3% reported from the 2018 national surveillance data from Ghana [1, 23]. The setting in the South Africa study was a formal gold mine employment with a well-functioning TB control program that was keeping track of drug resistant TB. In contrast, but for the outreach program to these mining communities, it is not possible to tell how long it would have taken for these drug resistant cases to be detected. This highlights the importance of mainstreaming TB screening services for such high risk populations [24].

In general males are more at risk for TB in Ghana and other countries and our study findings are in line with this [17,24]. It is reported that in illegal artisanal small-scale mining settings

in Ghana, women may constitute up to 50% of the labour force and play various roles [22,25,26]. The men however undertake more hazardous activities including underground mining which may expose them more to silica increasing the risk for TB [18,25-27].

TB prevalence surveys have shown chest X-rays to be more sensitive than symptom screening for TB detection since some people with TB may not have symptoms [28]. In our study the sensitivity and specificity of chest x-ray as a screening tool for TB fell within the range of what has been reported [29]. Symptom screening, particularly cough of any duration, on the other hand is reported to be less sensitive with pooled sensitivity and specificity in the range of 40 to 74% and 69 to 90% respectively [29]. Several studies on the diagnostic value of x-ray and symptoms screening for TB case detection have been conducted in different populations and among miners but there’s a dearth of such studies in mining communities to compare with [5, 30-34]. Comparing across such studies may however not be straightforward because of the different modalities of symptom definitions, screening criteria and subjectivity in reading of X-rays. [33,35] In our study the sensitivity (86.3%) of cough of any duration as a screening tool for the detection of TB was relatively high and even ranked higher than that of chest X-ray even though it was comparable. The combination of cough and X-ray raised the sensitivity to 95%. This finding is similar to what van’t Hoog and colleagues reported in a prevalence study in Kenya even though the cough in their study was 2 weeks or more in duration [33].

In this TB case detection outreach to the mining communities, those who were eligible to produce sputum for testing consisted of those who had an X-ray suggestive of TB, symptoms suggestive of TB and those who for some reason could not take X-ray totalling about 23% of the screened population. This is relatively higher compared to the 13% identified during the TB prevalence survey [17]. The difference may be related to the expertise in reading the X-rays (radiologists in the prevalence survey as opposed to physician assistants in our survey)

and the cough criteria (2 weeks cough in the prevalence survey). The yield of TB cases was however much higher and may justify the use of this approach of combining symptom and x-ray screening used in the mining communities as opposed to a sequential algorithm of symptom screening following by X-ray. Further studies assessing a cost benefit analyses and factoring in yield of TB cases may shed more light on the more beneficial and cost-effective approach to use in high risk populations in hard to reach communities.

This study has a number of limitations which include the point that the diagnosis of TB was based on results from GeneXpert to the exclusion of those who may have had clinically diagnosed TB. Secondly, having used routine data already collected, there was no control over standardizing the measurement and reporting of variables to enable further analyses of interest nor was it not possible to apply quality control measures or checks. For example, in the data set the recording cough of any duration without always specifying the duration for each participant limited the ability to assess the sensitivities of the different durations of cough and the diagnosis of TB. It is therefore being recommended to the NTP to have standardized data collection tools for all teams engaged in outreach programs to enable optimum analyses of data to inform programming. Finally, with this study being conducted in mining communities the findings may not be generalizable to other communities in the country.

Conclusion

The high risk of TB in the mining communities and in miners as shown in this study reinforces the need to target these populations with outreach programs particularly given that they may be in hard to reach areas. Even though the combination of cough and chest X-ray had the highest sensitivity, the diagnostic value of cough highlights the usefulness of symptom screening in this population that may be harnessed even in the absence of X-ray to identify those suspected to have TB for TB diagnostic evaluation.

Acknowledgement

The authors acknowledge with much appreciation the staff of the Ghana National Tuberculosis Control Program, regional health directorates of Ashanti, Brong Ahafo and Western Regions, the staff of the district health directorates of the participating districts, the communities that were screened and the various screening teams for the diverse roles played in the TB case finding exercise. Their efforts enabled the generation of the data for this study. Dr Rafiq Okine, Mr Fred Osei-Sarpong and Mr Felix Sorvor are also appreciated for the technical support.

REFERENCES

1. World Health Organization. Global tuberculosis report 2019. 2019 https://apps.who.int/iris/bitstream/handle/10665/329368/9789241565714-eng.pdf?ua=1
2. Ghana Health Service/Ministry of Health. The National Tuberculosis Health Sector Strategic Plan for Ghana 2015–2020. Accra, Ghana. https://www.ccmghana.net/images/PRs/NTP/TB-health-sector-plan-2015-2020.compressed.pdf
3. Stop TB Partnership. Ghana Tuberculosis (TB) situation in 2018 http://www.stoptb.org/resources/cd/GHA_Dashboard.html
4. World Health Organization. The End TB Strategy Global strategy and targets for tuberculosis prevention, care and control after 2015. 2015. http://www.who.int/tb/strategy/End_TB_Strategy.pdf
5. Den Boon S, Verver S, Lombard CJ, Bateman ED, Irusen EM, Enarson DA, et al. Comparison of symptoms and treatment outcomes between actively and passively detected tuberculosis cases: the additional value of active case finding. Epidemiol Infect. 2008; 136:1342–1349.
6. Golub JE, Mohan CI, Comstock GW, Chaisson RE. Active case finding of tuberculosis: historical perspective and future prospects. Int J Tuberc Lung Dis. 2005; 9(11):1183–1203.
7. Kranzer K, Houben RMGJ, Glynn JR, Bekker LG, Wood R, Lawn SD. Yield of HIV-associated tuberculosis during intensified case finding in resource-limited settings: a systematic review and meta-analysis. Lancet Infect Dis 2010;10:93–102.
8. Ghana Health Service/Ministry of Health. End-Term Comprehensive External Review Report of Ghana National Tuberculosis Health Sector Strategic Plan for Ghana 2009 – 2013. 2013.
9. Stuckler D, Basu S, McKee M, Lurie M. Mining and risk of tuberculosis in Sub-Saharan Africa Am J Public Health. 2011;101:524–530. doi:10.2105/AJPH.2009.175646
10. Stop TB Partnership TB Reach. Improving tuberculosis case detection; A compendium of TB REACH case studies, lessons learned and a monitoring and evaluation framework. 2015 http://www.stoptb.org/assets/documents/resources/publications/technical/TB_Case_Studies.pdf

11. Tuberculosis among miners, families and communities in Myanmar http://www.searo.who.int/myanmar/areas/tb_amongcommunities/en/ Accessed 6 March 2018 http://origin.searo.who.int/myanmar/areas/tuberculosisarchives/en/
12. Myint O, Saw S, Isaakidis P, Mohammed Khogali M, Hoa NB. et al. Active case-finding for tuberculosis by mobile teams in Myanmar: yield and treatment outcomes. Infectious Dis Poverty 2017; 6:77 doi 10.1186/s40249-017-0291-5
13. Churchyard G J, Kleinschmidt I, Corbett EL, Murray J, Smit J, De Cock KM. Factors associated with an increased case-fatality rate in HIV-infected and non-infected South African gold miners with pulmonary tuberculosis. Int J Tuberc Lung Dis 2000; 4(8):705-12.
14. Global Fund to fight HIV, TB and Malaria TB and HIV. Ghana HIV and TB Concept Note: Investing for impact against tuberculosis and HIV 2014. https://data.theglobalfund.org/investments/documents/GHA
15. STOP TB Partnership. TB Key Populations Brief: Miners. 2015. http://www.stoptb.org/assets/documents/resources/publications/acsm/KP_Miners_Spreads.pdf
16. STOP TB Partnership. Data for Action for Tuberculosis Key, Vulnerable and Underserved Populations. 2017. http://www.stoptb.org/assets/documents/communities/Data%20for%20Action%20for%20Tuberculosis%20Key,%20Vulnerable%20and%20Underserved%20Populations%20Sept%202017.pdf
17. Bonsu F, Addo KK, Alebachew Z, Gyapong J, Badu-Peprah A, Gockah R, et al. National population-based tuberculosis prevalence survey in Ghana 2013. Int J Tuberc Lung Dis. 2020 Mar 1;24(3):321-328. doi: 10.5588/ijtld.19.0163
18. Ngosa K, and Naidoo RN. The risk of pulmonary tuberculosis in underground copper miners in Zambia exposed to respirable silica: a cross-sectional study. BMC Public Health 2016: 16:855 doi: 10.1186/s12889-016-3547-2
19. Naidoo RN, Robins TG, Murray J. Respiratory outcomes among South African coal miners at autopsy. Am J Ind Med. 2005;48(3):217–24.
20. Adjei S, Oladejo NK, Adetunde IA. The impact and effect of illegal mining (galamsey) towards the socio-economic development of mining communities: a case study of Kenyasi in the Brong Ahafo Region. Int. J. Modern Soc. Sci.2012; 1(1): 38-55

http://modernscientificpress.com/Journals/ViewArticle.aspx?YTDXIp8pwb35qABc+2BV/2sxro7nTbAPwEKec1E3+qjxSterX62iOlFJYQs0xAkr

21. Owusu-Nimo F, Mantey J, Nyarko KB, Appiah-Effah E, Aubynn A. Spatial distribution patterns of illegal artisanal small-scale gold mining (*Galamsey*) operations in Ghana: A focus on the Western Region. Heliyon 4 (2018) e00534. doi: 10.1016/j.heliyon.2018.e00534

22. Hinson G. A Contextual Review of the Ghanaian Small-scale Mining Industry. Mining minerals and sustainable Development 2001 No 76 https://pubs.iied.org/pdfs/G00722.pdf

23. Calver AD, Falmer AA, Murray M, Strauss OJ, Streicher EM, Hanekom M, et al. Emergence of increased resistance and extensively drug-resistant tuberculosis despite treatment adherence, South Africa. Emerg Infect Dis 2010 Feb; 16:264 doi: 10.3201/eid02l0.090968

24. Saidu IA, Nasir Z, Goni BW. Social determinants of tuberculosis in sub-Saharan Africa: A systematic review GJMEDPH 2014; Vol. 3, issue 4 1-14 https://pdfs.semanticscholar.org/dda7/d6932ee4e127cb418e55cca1eb49328cd055.pdf?_ga=2.103536320.1373454180.1598103518-1107993822.1598103518

25. McQuilken, J and Hilson, G Artisanal and small-scale gold mining in Ghana. Evidence to inform an ‘action dialogue’. International Institute for Environment and Development, London. 2016. http://pubs.iied.org/16618IIED

26. Yakovleva N. Perspectives on female participation in artisanal and small-scale mining: A case study of Birim North District of Ghana. Resources Policy2007; 32: 29–41 https://www.womin.org.za/images/impact-of-extractive-industries/women-and-artisanal-mining/N%20Yakovleva%20-%20Female%20Participation%20in%20ASM%20Ghana.pdf

27. Chanda-Kapata P, Osei-Afriyie D, Mwansa C, Kapata N. Tuberculosis in the mines of Zambia: A case for intervention. Asian Pac J Trop Biomed 2016; 6(9): 803–807 http://dx.doi.org/10.1016/j.apjtb.2016.06.015

28. World Health Organization. Chest radiography in tuberculosis detection – summary of current WHO recommendations and guidance on programmatic approaches. 2016. https://apps.who.int/iris/bitstream/handle/10665/252424/9789241511506-eng.pdf;jsessionid=D01EEF4F066E475A487CBB80CC31C1C5?sequence=1

29. World Health Organization Systematic screening for active tuberculosis: principles and recommendations. 2013. https://www.who.int/tb/publications/Final_TB_Screening_guidelines.pdf

30. Day JH, Charalambous S, Fielding KL, Hayes RJ, Churchyard GJ, Grant AD. Screening for tuberculosis prior to isoniazid preventive therapy among HIV-infected gold miners in South Africa. Int J Tuberc Lung Dis 2006;10:523–529.
31. Lewis JJ, Charalambous S, Day JH, Fielding KL, Grant AD, Hayes RJ, et al. HIV infection does not affect active case finding of tuberculosis in South African gold miners. Am J Respir Crit Care Med. 2009; 180: 1271–1278. Epub 2009 Sep 1210.
32. Morasert T, Worapas W, Kaewmahit R, Uphala W. Prevalence and risk factors associated with tuberculosis disease in Suratthani Central Prison, Thailand 2018; 22(10):1203–1209 http://dx.doi.org/10.5588/ijtld.17.0654
33. van't Hoog AH, Meme HK, Laserson KF, Agaya JA, Muchiri BG, Githui WA, et al. Screening strategies for tuberculosis prevalence surveys: the value of chest radiography and symptoms. PLoS ONE 2012; 7(7): e38691. doi:10.1371/journal.pone.0038691
34. Nguyen DT, Bang ND, Hung NQ, Beasley RP, Hwang LY, Graviss EA. Yield of chest radiograph in tuberculosis screening for HIV-infected persons at a district-level HIV clinic. Int J Tuberc Lung Dis 2016; 20 (2):211–7. https://doi.org/10.5588/ijtld.15.0705
35. Creswell J, Qina ZZ, Gurungb R, Lamichhanec B, Yadavb DK, Prasaic MK, et al. The performance and yield of tuberculosis testing algorithms using microscopy, chest x-ray, and Xpert MTB/RIF. J Clin Tuberc Other Mycobact. Dis. 2019; 14: 141–6 https://doi.org/10.1016/j.jctube.2018.11.002

Supporting Information

S1 Fig. Fig 1. Map of Ghana showing districts in which case finding activities were undertaken in selected mining communities from May 2017 to January 2018. Fig 1 legend shows the range of the number of TB cases detected per 1000 persons screened from the TB case finding exercise in the respective districts

S2 Fig. Fig 2. Flow chart of TB case finding screening exercise undertaken in mining communities in 21 districts in Ghana from May 2017 to January 2018

S1 File. Data set for TB case finding activities

Figure

Click here to access/download;Figure;Figure 1 Map of Ghana districts TB case finding.tif

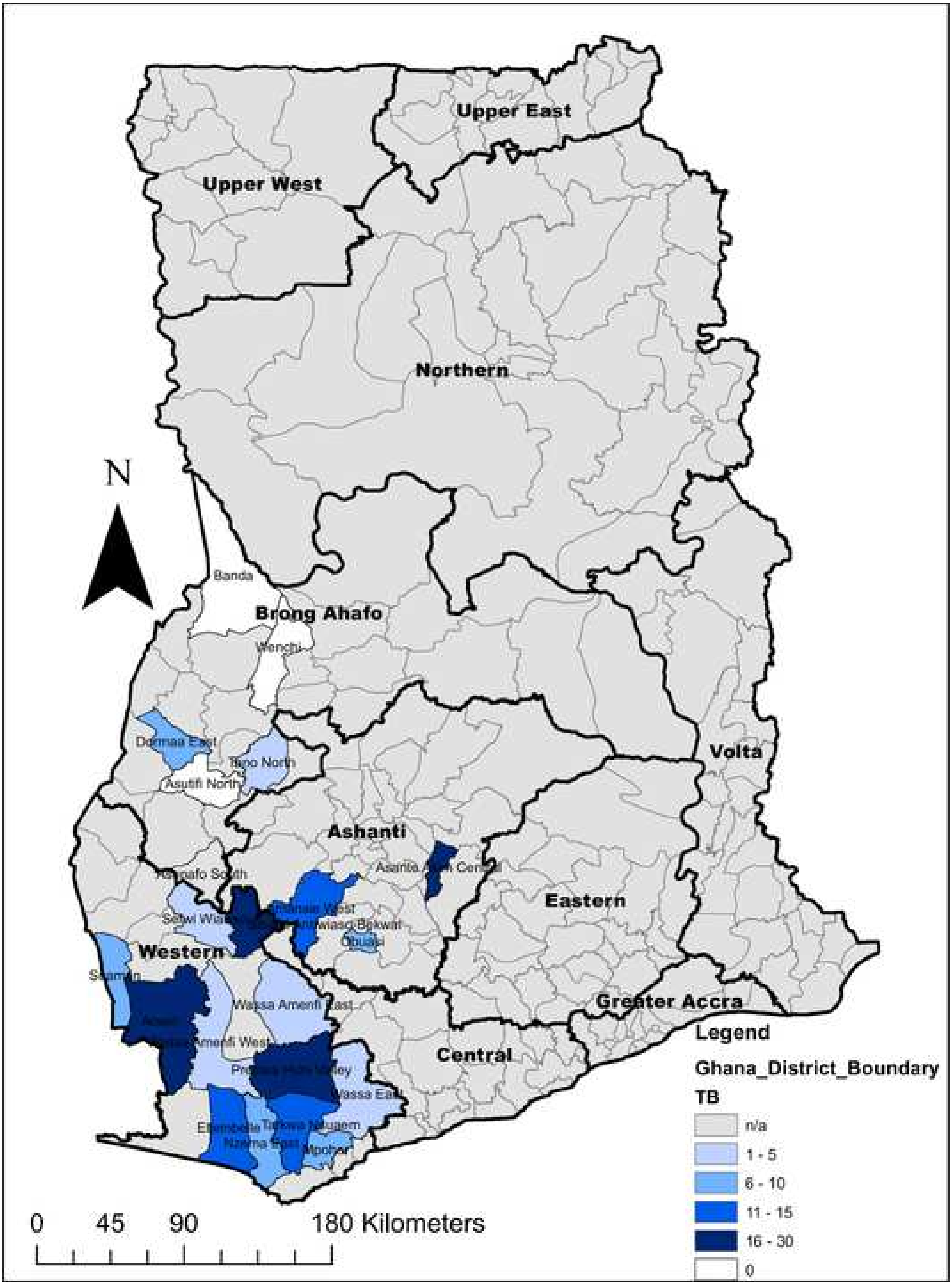

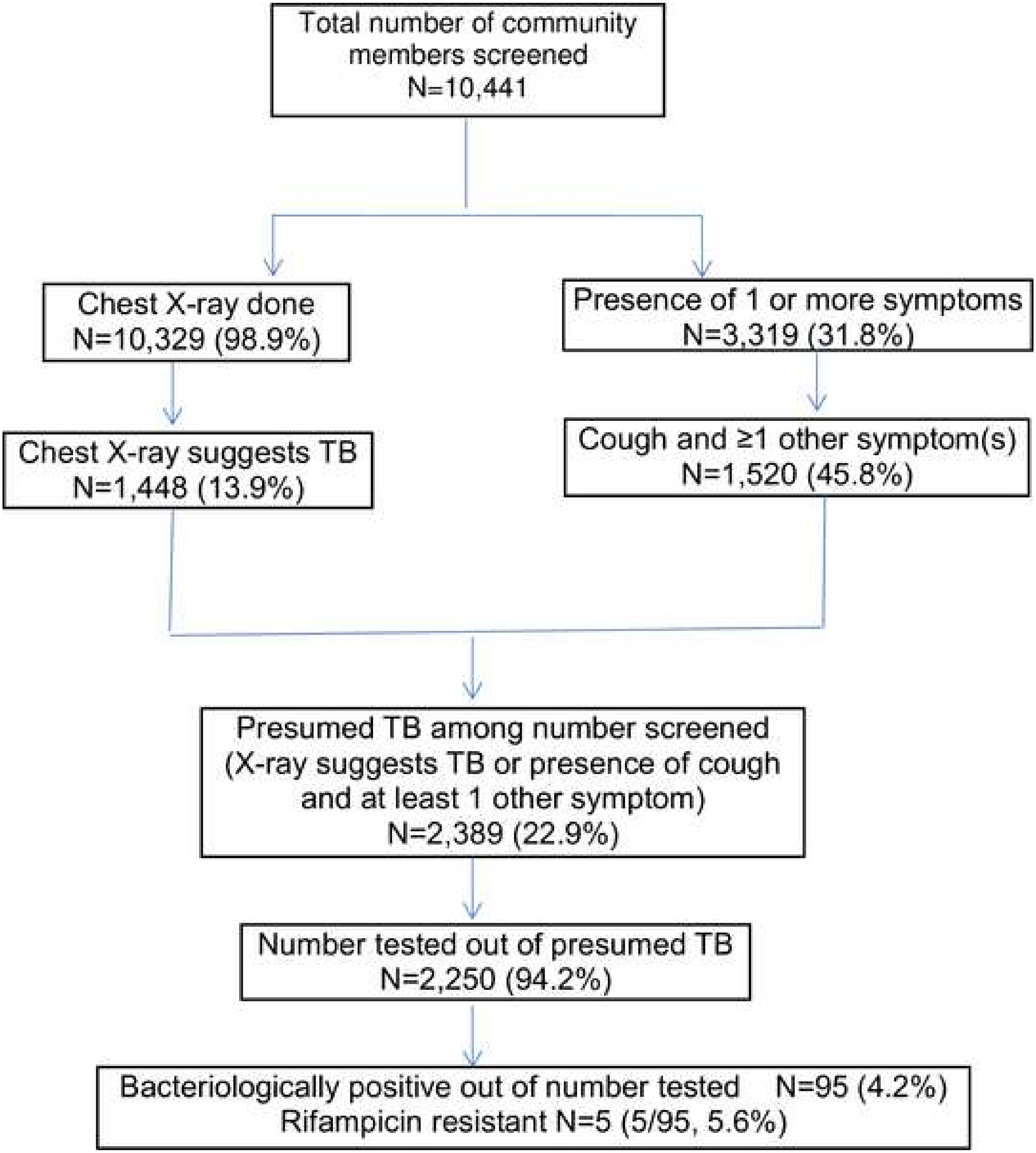
Total number of community members screened N=10,441
Chest X-ray done N=10,329 (98.9%)
Presence of 1 or more symptoms N=3,319 (31.8%)
Chest X-ray suggests TB N=1,448 (13.9%)
Cough and ≥1 other symptom(s) N=1,520 (45.8%)
Presumed TB among number screened (X-ray suggests TB or presence of cough and at least 1 other symptom) N=2,389 (22.9%)
Number tested out of presumed TB N=2,250 (94.2%)
Bacteriologically positive out of number tested N=95 (4.2%)
Rifampicin resistant N=5 (5/95, 5.6%)

Click here to access/download
Supporting Information
Data Set TB in Mining Communities Ghana 28 October 20.xlsx
