# Supplementary material for: Case finding of tuberculosis among mining communities in Ghana
Source: PLoS One. 2021 Mar 18;16(3):e0248718. doi: 10.1371/journal.pone.0248718 (PMC7971527; doi:10.1371/journal.pone.0248718)
Supplement: Attachment — Submitted filename: Review.docx [file pone.0248718.s004.docx]

Authors have amended the manuscript satisfactorily according to the recommendations provided in the first review. I, therefore, recommend its publication.

Shamim Mohammad
